# A Combination of Cardamonin and Doxorubicin Selectively Affect Cell Viability of Melanoma Cells: An In Vitro Study

**DOI:** 10.3390/antiox13070864

**Published:** 2024-07-19

**Authors:** Lara Ebbert, Claudia von Montfort, Chantal-Kristin Wenzel, Andreas S. Reichert, Wilhelm Stahl, Peter Brenneisen

**Affiliations:** Institute of Biochemistry and Molecular Biology I, Medical Faculty and University Hospital Düsseldorf, Heinrich Heine University Düsseldorf, 40225 Düsseldorf, Germanychantal-kristin.wenzel@hhu.de (C.-K.W.);

**Keywords:** melanoma, cardamonin, chalcone, doxorubicin, apoptosis, cytotoxicity, selectivity, ROS, mitochondria

## Abstract

Treatment of the most aggressive and deadliest form of skin cancer, the malignant melanoma, still has room for improvement. Its invasive nature and ability to rapidly metastasize and to develop resistance to standard treatment often result in a poor prognosis. While the highly effective standard chemotherapeutic agent doxorubicin (DOX) is widely used in a variety of cancers, systemic side effects still limit therapy. Especially, DOX-induced cardiotoxicity remains a big challenge. In contrast, the natural chalcone cardamonin (CD) has been shown to selectively kill tumor cells. Besides its anti-tumor activity, CD exhibits anti-oxidative, anti-inflammatory and anti-bacterial properties. In this study, we investigated the effect of the combinational treatment of DOX with CD on A375 melanoma cells compared to normal human dermal fibroblasts (NHDF) and rat cardiac myoblasts (H9C2 cells). DOX-induced cytotoxicity was unselective and affected all cell types, especially H9C2 cardiac myoblasts, demonstrating its cardiotoxic effect. In contrast, CD only decreased the cell viability of A375 melanoma cells, without harming normal (healthy) cells. The addition of CD selectively protected human dermal fibroblasts and rat cardiac myoblasts from DOX-induced cytotoxicity. While no apoptosis was induced by the combinational treatment in normal (healthy) cells, an apoptosis-mediated cytotoxicity was demonstrated in A375 melanoma cells. CD exhibited thiol reactivity as it was able to directly interact with N-acetylcysteine (NAC) in a cell-free assay and to induce heme oxygenase-1 (HO-1) in all cell types. And that took place in a reactive oxygen species (ROS)-independent manner. DOX decreased the mitochondrial membrane potential (Δψ_m_) in all cell types, whereas CD selectively decreased mitochondrial respiration, affecting basal respiration, maximal respiration, spare respiratory capacity and ATP production in A375 melanoma cells, but not in healthy cardiac myoblasts. The DOX-induced cytotoxicity seen in melanoma cells was ROS-independent, whereas the cytotoxic effect of CD was associated with CD-induced ROS-formation and/or its thiol reactivity. This study highlights the beneficial properties of the addition of CD to DOX treatment, which might protect patients from DOX-induced cardiotoxicity. Future experiments with other tumor cell lines or a mouse model should substantiate this hypothesis.

## 1. Introduction

Malignant melanoma is the most aggressive and deadliest form of skin cancer [1,2,3]. Its ability to rapidly metastasize and its invasive growth result in a poor prognosis with a median overall survival rate of less than one year in patients with metastasized melanoma [4]. Successful treatment becomes more and more important as incidences are continuously increasing. More patients require an effective therapy [5,6,7]. Currently, chemotherapy is standard in most metastatic tumors. However, the therapeutic effectiveness is limited by multidrug resistances [6,8,9]. The developing insensitivity to a variety of different, structurally unrelated therapeutic drugs with different mechanisms of action may be caused by the tumor’s ability to alter cell cycle checkpoints, to impair apoptosis and autophagy or to lower the intracellular drug accumulation [8,9,10]. Especially in melanomas, the efficacy of conventional chemotherapy is limited, as these cells often exhibit drug resistance [3,5,8,11]. Next to chemotherapy, malignant melanomas also show a high resistance to immunotherapy and to new generation tumor-targeted drugs such as BRAF inhibitors [1,2,5,11]. Generally, drug resistance may be intrinsic or develop over time in the course of therapy [1,11]. The precise reason for drug resistance in melanoma is still unknown. Nevertheless, regulation of apoptosis is suggested to play an important role as melanoma cells express less pro-apoptotic molecules and exhibit altered apoptotic pathways [11].

The anthracycline derivative doxorubicin (DOX) is a highly effective chemotherapeutic drug and is clinically used against a variety of tumors [10,12]. Its mode of action is complex, including DNA intercalation, inhibition of topoisomerase II, generation of reactive oxygen species (ROS), dysregulation of Ca^2+^ and iron homeostasis, the release of cytochrome c from mitochondria and the induction of apoptosis [10,12,13]. Despite its efficacy in tumor therapy, DOX treatment results in a variety of serious side effects, which range from nausea and severe headache to long-term adversity like cardio- and nephrotoxicity [10,12,13,14]. Numerous studies suggest that DOX might be effective to treat melanomas [7,15,16], while other studies report on intrinsic resistance to DOX, rendering it ineffective for therapy [1,8,17,18]. Currently, DOX is not clinically administered in human melanomas, but combinational approaches have been tested to increase treatment efficacy and circumvent resistance. For example, Frank et al. blocked the ATP-binding cassette (ABC) receptor ABCB5 which abrogated the ABCB5-mediated DOX efflux, thereby significantly enhancing intracellular drug accumulation and reversing DOX resistance in G3361 melanoma cells [8]. However, circumventing resistance may not prevent negative side effects, unselective drug distribution and systemic toxicity which still remain a big challenge in DOX therapy [12,13,14]. As an in situ approach, DOX treatment decreased tumor growth while mitigating systemic side effects [6]. Another possible approach is to use different drug delivery systems. In this regard, Lima et al. combined DOX with calcium phosphate nanoparticles functionalized with hyaluronic acid which targeted tumor cells and, thus, significantly decreased the IC_50_ in A375 melanoma cells compared to a DOX single treatment [7]. Furthermore, combinational approaches with inhibitors, miRNAs, other chemotherapeutic drugs and natural compounds have been described [2,12,13]. The addition of redox-active cerium oxide nanoparticles (CNP) to DOX treatment enhanced its cytotoxicity, ROS production and oxidative damage in melanoma cells, while not harming and even protecting normal dermal fibroblasts (NHDF) [19]. In a completely different approach, Rok et al. showed that the IC_50_ values were lower in COLO829 and A375 melanoma cells compared to human dermal fibroblasts and epidermal melanocytes with the use of the antibiotic tigecycline (TGC), but only in a rather narrow concentration range [20]. The combination of DOX with the chalcone naringenin, occurring in citrus plants, reversed the DOX-induced upregulation of inflammatory molecules and downregulation of antioxidant enzymes in vivo [21]. Generally, the application of natural compounds like chalcones has gained increasing attention in cancer therapy [22,23,24]. Chalcones are α,β-unsaturated ketones, which belong to the family of flavonoids and are often responsible for the yellow pigmentation of plants [25,26,27]. Natural chalcones have been reported to have anti-tumor, anti-oxidative, anti-inflammatory and anti-bacterial activities [25,26,27,28,29].

One example is the natural chalcone cardamonin (CD), which occurs in cardamom spice and can be extracted from *Alpinia* species [27,28]. It has been shown that CD influences the nuclear factor-κB (NF-κB) pathway, which controls DNA transcription, regulates immune responses to infections and plays a key role in neoplastic processes [30,31,32]. A potential mechanism is the thiol reactivity of CD through its α,β-unsaturated carbonyl group, by which it can act as Michael acceptor and react with thiol groups of proteins [23,24]. CD prevented the growth and proliferation of multiple myeloma cell lines [32] and exhibited a protective effect from acute lung injury [33]. Moreover, CD led to increased generation of ROS in colorectal cancer cells and, consequently, ROS-mediated cytotoxicity [34]. In SH-SY5Y human neuroblastoma cells, CD altered the mitochondrial membrane potential (Δψ_m_), induced caspase activity and apoptotic cell death, while not affecting NHDF and, thus, indicating selective toxicity [35]. In the same context, CD selectively decreased proliferation, lowered the invasive capacity, and exhibited cytotoxic effects on A375 melanoma cells [36]. Furthermore, CD was demonstrated to protect the heart from oxidative damage, apoptosis and inflammatory injury caused by DOX in vitro and in vivo [29]. However, while CD could be valuable to prevent DOX-induced cardiotoxicity, Qi et al. did not demonstrate the effectiveness of the combinational treatment in tumor therapy [29]. In this study, we focused on the combinational treatment of DOX and CD, showing its efficacy on A375 human melanoma cells, while protecting NHDF and rat cardiac myoblasts from DOX-induced cytotoxicity.

## 2. Materials and Methods

### 2.1. Materials

All chemicals were purchased from Merck (Darmstadt, Germany), Sigma-Aldrich (St. Louis, MO, USA), Roth (Karlsruhe, Germany) or Thermo Fisher Scientific (Waltham, MA, USA), unless stated otherwise. Doxorubicin (DOX), penicillin–streptomycin, bromophenol blue, acetic acid and sodium dihydrogen phosphate were obtained from Merck. Cardamonin (CD) and the pan caspase inhibitor Q-VD-OPh were purchased from MedChemExpress (Monmouth Junction, NJ, USA). Dulbecco’s Modified Eagle’s Medium (DMEM), dimethyl sulfoxide (DMSO) for substance dissolution, phosphate buffered saline (PBS), 3-(4,5-dimethylthiazol-2-yl)-2,5-diphenyltetrazolium bromide (MTT), carbonyl cyanide 3-chlorophenylhydrazone (CCCP), staurosporine (Sts), protease inhibitor cocktail [37], 2-mercaptoethanol, PageRuler^TM^ Prestained Protein Ladder, sulforhodamine B (SRB), trichloroacetic acid (TCA), 2′,7′-dichlorofluorescein diacetate (H_2_DCF-DA), 6-hydroxy-2,5,7,8-tetramethylchroman-2-carboxylic acid (trolox), catalase−polyethylene glycol (PEG-catalase) and N-acetylcysteine (NAC) were obtained from Sigma-Aldrich. GlutaMax^TM^, tetramethylrhodamine methyl ester (TMRM) and Hank’s Balanced Salt Solution (HBSS) were purchased from Thermo Fisher Scientific, and fetal bovine serum (FBS) from PAN-Biotech GmbH (Aidenbach, Germany). DMSO for MTT assay, sodium dodecyl sulfate (SDS), glycerol and Tris base were obtained from Roth. Z-VAD-FMK was purchased from AdooQ BioScience (Irvine, CA, USA), and Clarity™ Western ECL Substrate and DC protein assay from Bio-Rad Laboratories, Inc. (Hercules, CA, USA). All reagents for the Mito Stress Test, including Seahorse XF Cell Mito Stress Test Kit (Cat. 103015-100), Seahorse XF DMEM Medium (pH 7.4, phenol red-free, 5 mM HEPES) and Seahorse XF Calibrant, were obtained from Agilent Technologies (Santa Clara, CA, USA).

### 2.2. Cell Culture

Human melanoma cell line A375 (ATCC^®^ CRL-1619) was purchased from the American Type Culture Collection (ATCC, Manassas, VA, USA). H9C2 rat cardiac myoblasts (H9C2 (2-1), 88092904) were obtained from Sigma-Aldrich and normal human dermal fibroblasts (NHDF, C-12300) were purchased from PromoCell (Heidelberg, Germany). All cell types were cultured in low glucose Dulbecco’s Modified Eagle’s Medium (DMEM, 1000 mg/L glucose), supplemented with 10% fetal bovine serum (FBS), streptomycin (100 μg/mL), penicillin (100 U/mL) and GlutaMAX™ (2 mM) at 37 °C in 5% CO_2_. Subconfluent cells (70–80% confluency) and high glucose (HG, 4500 mg/L) DMEM without FBS was used for all experiments.

### 2.3. Cell Viability Assay

The cell viability was measured by the MTT (3-(4,5-dimethylthiazol-2-yl)-2,5-diphenyltetrazolium bromide) assay, which is based on the conversion of MTT to a purple formazan dye. As this reaction is performed by mitochondrial dehydrogenases, the assay reflects the mitochondrial activity of still living cells. Briefly, subconfluent cells in 48-well plates were either mock-treated (0.5% DMSO) or treated with different concentrations of CD and DOX alone for 24 to 96 h or in combination for 96 h. After treatment, cells were washed with phosphate buffered saline (PBS) and incubated with MTT solution (0.5 mg/mL in HG DMEM) for 30 min (A375) or 2 h (H9C2 and NHDF). Thereafter, MTT solution was removed, cells were washed with PBS and the intracellular formazan was extracted using DMSO (200 µL/well). Subsequently, absorbance was measured using the FLUOstar OPTIMA plate reader (BMG Labtech, Ortenberg, Germany). Absorbance of DMSO served as blank and mock-treated control was set to 100%.

### 2.4. Mitochondrial Membrane Potential (Δψ_m_)

The mitochondrial membrane sensitive and red fluorescent probe tetramethylrhodamine methyl ester (TMRM) was used to measure the mitochondrial membrane potential (Δψ_m_) via flow cytometry. In normal (healthy) cells with active/intact mitochondria and a physiological Δψ_m_, TMRM accumulates and a red fluorescent signal can be detected. With the induction of apoptosis or treatment with uncoupling agents like carbonyl cyanide 3-chlorophenylhydrazone (CCCP), the mitochondrial membrane depolarizes and the TMRM signal decreases. Briefly, cells were either mock-treated (0.5% DMSO) or treated with 5 µM CD and 0.5 µM DOX alone or in combination for 2 or 4 h. For the positive control, 10 μM of the oxidative phosphorylation uncoupler CCCP was used and 1 µM of DOX served as the DOX-positive control. After 2 and 4 h, respectively, TMRM solution was added to reach a final concentration of 100 nM. After staining, cells were washed with and resuspended in Hank’s Balanced Salt Solution (HBSS) and the level of TMRM was determined by flow cytometry using a CyFlow Cube 6 (Sysmex Corporation, Kobe, Japan). An excitation wavelength of 488 nm and an emission wavelength of 590 ± 25 nm were chosen. FlowJo 10.8.1 software (BD Biosciences, Franklin Lakes, NJ, USA) was used to analyze data and calculate the median fluorescent intensity (MFI). The MFI of unstained cells served as the negative control and the mock-treated control was set to 100%.

### 2.5. Intracellular ROS

The level of reactive oxygen species (ROS) was measured using 2′,7′-dichlorofluorescein diacetate (H_2_DCF-DA) via flow cytometry. The cell permeable, non-fluorescent agent H_2_DCF-DA is a chemically reduced form of fluorescein. Within the cell, it is de-esterified into 2′,7′-dichlorofluorescin (DCF), which accumulates intracellularly and becomes highly fluorescent upon oxidation by ROS.

For the basal ROS measurement, cells were treated with 0.1 μM H_2_DCF-DA in HBSS for 20 min. After staining, the level of DCF was determined by flow cytometry using a CyFlow Cube 6. An excitation wavelength of 488 nm and an emission wavelength of 536 ± 20 nm were chosen. The FlowJo software was used for data analysis, to create histogram overlays and to calculate the MFI. The MFI of unstained cells served as negative control and the MFI of A375 cells was set to 100%. 

For measuring ROS after substance treatment, cells were treated with 20 μM H_2_DCF-DA in HBSS for 20 min and subsequently washed with and resuspended in HBSS. Afterwards, cells were either mock-treated (0.5 % DMSO) or treated with 5 µM CD and 0.5 µM DOX alone or in combination. A375 cells were treated for 15 min to 4 h, and H9C2 and NHDF for 1 h. After the treatment, the level of DCF was determined by flow cytometry as described above. The MFI of unstained cells served as negative control and the mock -treated control was set to 1.

### 2.6. Antioxidant Treatment/ROS Rescue Experiment

The effect of antioxidants on DOX and CD-induced ROS production was measured using the DCF assay as described above. Briefly, A375 cells were treated with 20 μM H_2_DCF-DA in HBSS for 20 min. After staining, the cells were washed with and resuspended in HBSS and either mock-treated or treated with antioxidants for 4 h. As antioxidants, 150 µM trolox and 4000 U/mL catalase alone or in combination, or 5 mM N-acetylcysteine (NAC) were used. Subsequently, the cells were either mock-treated (0.5% DMSO) or treated with 5 µM CD and 0.5 µM DOX alone or in combination for 1 h. After the treatment, the level of DCF was determined by flow cytometry as described above. The MFI of unstained cells served as the negative control and the mock-treated control was set to 100%.

### 2.7. NAC Cell Viability Rescue Experiment

NAC, a derivative of the natural amino acid L-cysteine, was used to perform a cell viability rescue experiment. For this, subconfluent cells in 48-well plates were mock-treated or preincubated with 5 mM NAC for 4 h. Subsequently, the cells were either mock-treated (0.5% DMSO) or treated with 5 µM CD and 0.5 µM DOX alone or in combination for another 24 h. After the treatment, the cell viability was quantified via the MTT assay as described above. The mock-treated control was set to 100%.

### 2.8. CD and NAC Interaction

The interaction of CD and NAC was analyzed in a cell-free assay using a UV/Vis spectrophotometer. The concentration of an organic substance containing a chromophore can be measured using UV/Vis spectroscopy. Cardamonin exhibits an absorption maximum at 340−390 nm, which is characteristic for chalcones [38]. A reduction at the absorption maximum is an indication that CD has undergone a reaction with NAC and less free CD can be detected. For the measurement of CD, a 50 μM CD solution in 0.1 M sodium dihydrogen phosphate buffer was incubated with 0.5 to 100 mM NAC for 10 min. Subsequently, the absorption between 250 and 500 nm was measured using the Photometer Ultrospec 1000/3000 (Pharmacia Biotech, Freiburg, Germany). The absorption spectrum of the buffer was used for background correction.

### 2.9. SDS-PAGE and Western Blotting

For protein detection and quantification, sodium dodecyl sulfate polyacrylamide gel electrophoresis (SDS-PAGE) and Western blotting were performed. For heme oxygenase-1 (HO-1) detection, subconfluent cells in 10 cm dishes were either mock-treated (0.5% DMSO) or treated with 5 µM CD for 4 to 24 h. For detection of cleaved PARP, subconfluent cells in 10 cm dishes were either mock-treated (0.5% DMSO) or treated with 5 µM CD and 0.1 or 0.5 µM DOX alone or in combination for 24 h. For the positive control, 20 μM of the protein kinase inhibitor staurosporine (Sts), a well-known inducer of apoptosis [39], was used and 1 µM DOX served as the DOX-positive control. After treatment, adherent and detached cells were lysed in SDS lysis buffer (1% SDS supplemented with 0.1% protease inhibitor cocktail (PIC)) and sonicated for 10 s, and the protein concentration of the lysates was determined using the DC™ Protein Assay Kit (Bio-Rad Laboratories, Hercules, CA, USA) according to the manufacturer’s protocol. For each sample, 20 µg of protein was mixed with one quarter volume of 4× Laemmli buffer (40% glycerol, 20% 2-mercaptoethanol, 12% SDS, 0.4% bromophenol blue), heated at 95 C for 10 min and applied to 12% SDS-polyacrylamide gels. The proteins were transferred onto a polyvinylidene fluoride (PVDF) membrane (GE Healthcare, Chicago, IL, USA) and, after primary and secondary antibody staining, visualized via enhanced chemiluminescence (ECL) using the Fusion-FX7 EDGE imaging system (Vilber Lourmat, Collégien, France). Protein quantification was performed using the software FusionCapt Advance FX 16.09 (Vilber Lourmat, Collégien, France). Monoclonal anti-HO-1 (1:1000, ADI-OSA-110, Enzo Biochem, Farmingdale, NY, USA), monoclonal anti-β-Tubulin (1:1000, #2128, Cell Signaling Technology, Danvers, MA, USA) and polyclonal anti-PARP (1:1000, #9542, Cell Signaling Technology, Danvers, MA, USA) were used as primary antibodies. Goat anti-rabbit IgG (1:10,000, #111–035-144, Jackson ImmunoResearch Labs, West Grove, PA, USA) and goat anti-mouse IgG (1:10,000; #ab97023, abcam, Cambrigde, UK) were used as secondary antibodies. β-Tubulin served as loading control.

### 2.10. Mito Stress Test

The mitochondrial respiration was measured using the Seahorse XF Cell Mito Stress Test (Agilent Technologies, Santa Clara, CA, USA) via the Seahorse XFe96 Analyzer (Agilent Technologies, Santa Clara, CA, USA) according to the manufacturer’s protocol. Based on the measurement of the oxygen consumption rate (OCR) and after the consecutive injection of mitochondrial stressors, different bioenergetic parameters including the basal respiration, maximal respiration, spare respiratory capacity and ATP production were calculated using Seahorse Wave 2.6.3 Desktop software (Agilent Technologies, Santa Clara, CA, USA). The following mitochondrial stressors were included in the Mito Stress Test kit: oligomycin (Oligo, final concentration: 2 µM), carbonyl cyanide-4 (trifluoromethoxy) phenylhydrazone (FCCP, final concentration: 0.5 µM for A375 and 1 µM for H9C2 cells) and rotenone/antimycin A (Rot/AA, final concentration: 0.5 µM). For this assay, 10,000 A375 cells/well or 18,000 H9C2 cells/well were seeded in a Seahorse XFe96 cell culture microplate and allowed to adhere overnight. Subsequently, the cells were either mock-treated (0.5% DMSO) or treated with 5 µM CD for 24 h. After addition of 0.5 µM DOX for the last 4 h of the CD treatment, the cells were washed with and resuspended in Seahorse XF DMEM Medium and placed at 37 °C in a non-CO_2_ incubator for 1 h. The sensor cartridge was hydrated in Seahorse XF Calibrant at 37 °C in a non-CO_2_ incubator overnight, and the mitochondrial stressors were added into the injection ports shortly before measurement. After calibration of the sensor plate, the cell culture microplate was placed in the Seahorse XFe96 analyzer and the OCR was measured. After the measurement, the cells were quantified by using the sulforhodamine B (SRB) assay. For SRB staining, the Seahorse Medium was removed, the cells were washed with PBS and fixed with 100 µL 10% trichloroacetic acid (TCA) per well for 1 h at 4 °C. Afterwards, the cells were washed five times with dH_2_O and dried at room temperature (RT). Subsequently, the cells were stained with 100 µL SRB solution (0.4% SRB in 1% acetic acid) per well for 15 min at RT, washed five times with 1% acetic acid and dried at RT. The dye was extracted in 100 µL 10 mM Tris base per well while gently rotating for 5 min at RT. The absorbance was measured at 492 nm and 620 nm (reference) using the Tecan Infinite 200 PRO microplate reader (Tecan Group AG, Männedorf, Switzerland). The Mito Stress Test raw data were normalized to the corresponding SRB staining and the mock-treated control was set to 100%.

### 2.11. Apoptosis Rescue Experiments

Firstly, the pan caspase inhibitors Z-VAD-FMK and Q-VD-OPh were used to investigate induction of apoptosis. Secondly, for the detection of PARP cleavage, subconfluent cells in 10 cm dishes were pretreated with 60 µM Z-VAD-FMK, followed by either a mock treatment (0.5% DMSO) or treatment with 5 µM CD and 0.5 µM DOX alone or in combination for another 24 h. PARP and cleaved PARP were visualized using SDS-PAGE and Western blotting as described above. For the rescue experiments, subconfluent cells in 48-well plates were pretreated with 100 µM Q-VD-OPh, followed by either a mock treatment (0.5% DMSO) or treatment with 5 µM CD and 0.5 µM DOX alone or in combination for another 24 h. Cell viability was assessed via the MTT assay as described above. The mock-treated control was set to 100%.

### 2.12. Statistical Analysis

Data were presented as mean ± standard error of the mean [40]. Mean values were calculated from at least three independent experiments unless stated otherwise. Statistical analysis was performed using the software Graph Pad Prism 9 (GraphPad Software, San Diego, CA, USA), with * *p* ≤ 0.05, ** *p* ≤ 0.01 and *** *p* ≤ 0.001 as levels of significance. One-way ANOVA with Dunnett’s multiple comparisons test was used to determine statistical significance in experiments with one independent variable (e.g., drug treatments compared to control). Two-way ANOVA with Sidak’s multiple comparisons test was used to determine statistical significance in experiments with two independent variables (e.g., comparing drug treatments between an antioxidant-untreated versus antioxidant-treated group). IC_50_ values after 96 h of treatment were calculated by non-linear curve fit analysis using Graph Pad Prism.

## 3. Results

Doxorubicin (DOX) is highly effective for treating a variety of carcinomas. However, severe side effects such as cardiomyopathies limit its therapeutic use [10,12,13,14]. Therefore, a combinational treatment with the natural chalcone cardamonin (CD) was studied, which earlier was shown to be non-toxic for normal (healthy) cells [35,36]. To prove treatment effectivity, skin melanoma cells (A375) were used as the representative tumor cell line. In contrast, for demonstrating selectivity, normal human dermal fibroblasts (NHDF) were used as a model for normal (healthy) cells, as fibroblasts are the most common cells in the connective tissue and are present in many other tissues. As the most prominent and severe side effect of DOX is cardiotoxicity, normal rat cardiac myoblasts (H9C2 cells) were studied as well. These cells have been used as they are energetically more similar to primary cardiomyocytes than, for example, mouse HL-1 cardiomyocytes [41].

### 3.1. CD Selectively Protected Normal (Healthy) Cells from DOX-Induced Cytotoxicity While Decreasing Cell Viability of Melanoma Cells

**Figure 1:**
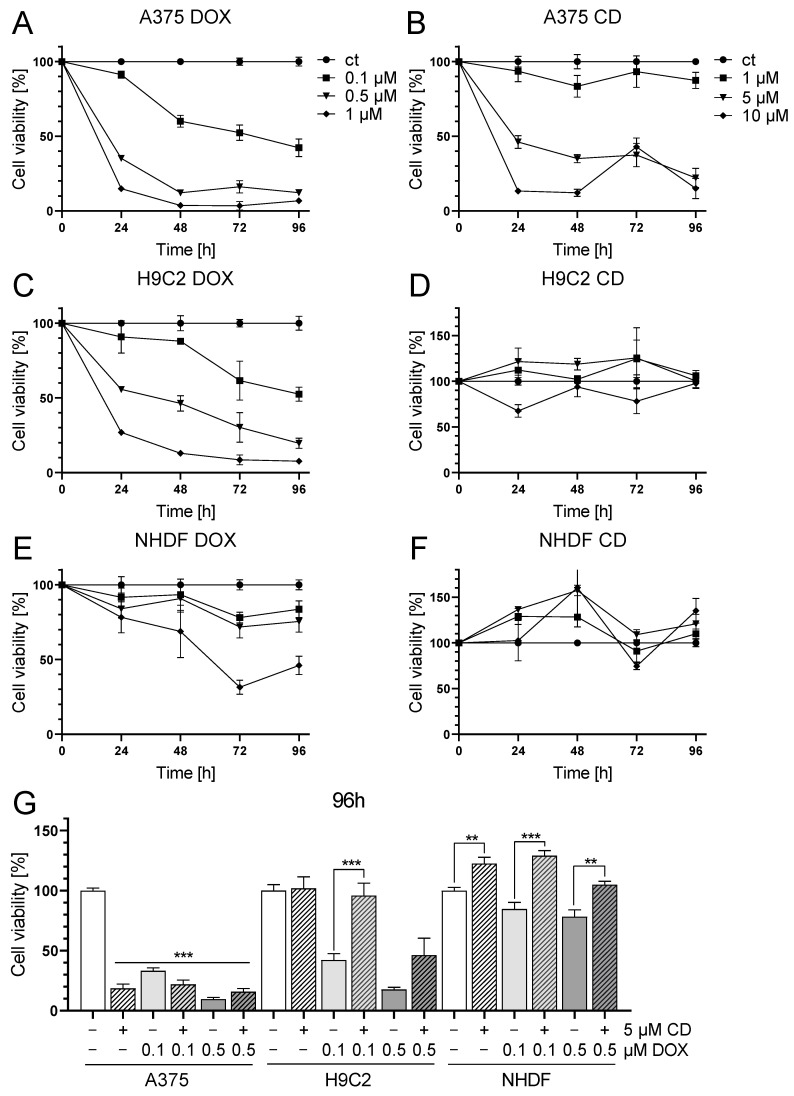
Effect of DOX and CD on the cell viability of A375 human melanoma cells, H9C2 rat cardiac myoblasts and normal human dermal fibroblasts (NHDF). (**A**–**F**): A375 (**A**,**B**), H9C2 (**C**,**D**) and NHDF (**E**,**F**) were treated with increasing concentrations of DOX (**A**,**C**,**E**) and CD (**B**,**D**,**F**) for 24, 48, 72 and 96 h. (**G**) For comparing A375, H9C2 and NHDF, cells were treated with different concentrations of DOX alone or in combination with CD for 96 h. Cell viability was determined via the MTT assay and mock-treated control (ct) was set to 100%. Data represent means ± SEM, *n* ≥ 3. (**G**) One-way ANOVA was used to determine statistical significance in A375 cells compared to control (white bar). Two-way ANOVA was used in H9C2 and NHDF to compare CD-untreated (0 µM) with CD-treated cells (5 µM). ** *p* ≤ 0.01, *** *p* ≤ 0.001.

First, single treatment with the chemotherapeutic agent DOX and the natural chalcone CD was tested in A375, H9C2 and NHDF. DOX significantly decreased cell viability in all cell lines in a time- and dose-dependent manner (Figure 1A,C,E), while CD selectively decreased the cell viability of A375 cells but not of the normal (healthy) cell strains H9C2 and NHDF (Figure 1B,D,F). 

With the highest concentration of 1 µM DOX, cell viability was decreased in A375 and H9C2 cells below 10% and below 50% in NHDF after 96 h of treatment. In A375 and H9C2 cells, even the lowest concentration of 0.1 µM DOX lowered the viability to about 50%. The highest concentration of 10 µM CD declined the cell viability to 15% in A375 cells after 96 h, while having no effect on both normal (healthy) cell strains.

The selectivity of both substances was best illustrated by the individual IC_50_ values (absolute IC_50_ values were calculated by a non-linear curve fit analysis after 96 h of treatment). When looking at DOX, the IC_50_ values were similar in A375 and H9C2 cells, with 0.08 µM in A375 and 0.12 µM in H9C2. The weakest effect of DOX was seen in NHDF with an IC_50_ value of 0.86 µM. In the case of CD treatment, IC_50_ values strongly differed between melanoma and normal (healthy) cells. CD was very effective in decreasing the cell viability of A375 cells, with an IC_50_ value of 2.76 µM, whereas normal (healthy) cells were less affected, with IC_50_ values of 17.86 µM for H9C2 and 18.51 µM for NHDF.

As cardiotoxicity was described to be the most prominent and severe side effect of DOX in cancer patients, which may even lead to acute heart failure, a combinational treatment with DOX and CD was tested to find out whether CD could protect cardiac myoblasts from DOX-induced toxicity (Figure 1G). Since DOX-induced cardiotoxicity in vivo is not an acute effect but emerges over time, a 96 h in vitro treatment was carried out. The selected treatment doses of DOX were 0.1 and 0.5 µM, as these concentrations already had a high impact on normal (healthy) cells. Regarding CD, a concentration of 5 µM was chosen, as no dose-dependent differences between 5 and 10 µM were observed in melanoma cells.

All single treatments as well as the combinational treatments significantly decreased cell viability in A375 cells compared to the mock-treated control (white bar). There were no significant differences between the single and the respective combinational treatment. In the healthy cardiac myoblasts, the DOX single treatment led to a significant decrease in cell viability compared to the mock-treated control, whereas the addition of 5 µM CD rescued the DOX-induced toxicity. In the case of 0.1 µM DOX, the addition of CD caused a significant increase in cell viability to the level of the mock-treated control. In the case of 0.5 µM DOX, the addition of CD increased cell viability from 17 to about 46%. Likewise, the cell viability of NHDF was significantly increased in the combinational treatment compared to the DOX single treatment, either to the level of the mock-treated control or above.

Thus, CD selectively protected H9C2 and NHDF from DOX-induced toxicity while decreasing the cell viability of A375 cells.

Concentrations of the combinational treatments (5 µM CD and 0.1 or 0.5 µM DOX, Figure 1G) were used for further studies. In some experiments, 1 µM DOX served as the DOX-positive control to reflect negative effects on normal (healthy) cells as well.

### 3.2. DOX Decreased the Mitochondrial Membrane Potential in A375, H9C2 and NHDF

Changes in mitochondrial membrane potential (Δψ_m_) may depend on increasing reactive oxygen species (ROS), which affect oxidative phosphorylation (OXPHOS) and finally result in cell death mechanisms [42]. Especially early targets of apoptosis have been described to be associated with a decrease in Δψ_m_ [43,44]. To study the effect of DOX and CD either alone or in combination on the Δψ_m_, the mitochondrial-membrane-sensitive and red fluorescent probe tetramethylrhodamine methyl ester (TMRM) was used to measure the Δψ_m_. Preliminary data revealed that between 15 min and 6 h, only 2 and 4 h DOX treatments decreased the Δψ_m_ in A375 cells. Therefore, 2 and 4 h time points were chosen for the following Δψ_m_ measurements. The uncoupler CCCP served as the positive control as it decreases the Δψ_m_.

**Figure 2:**
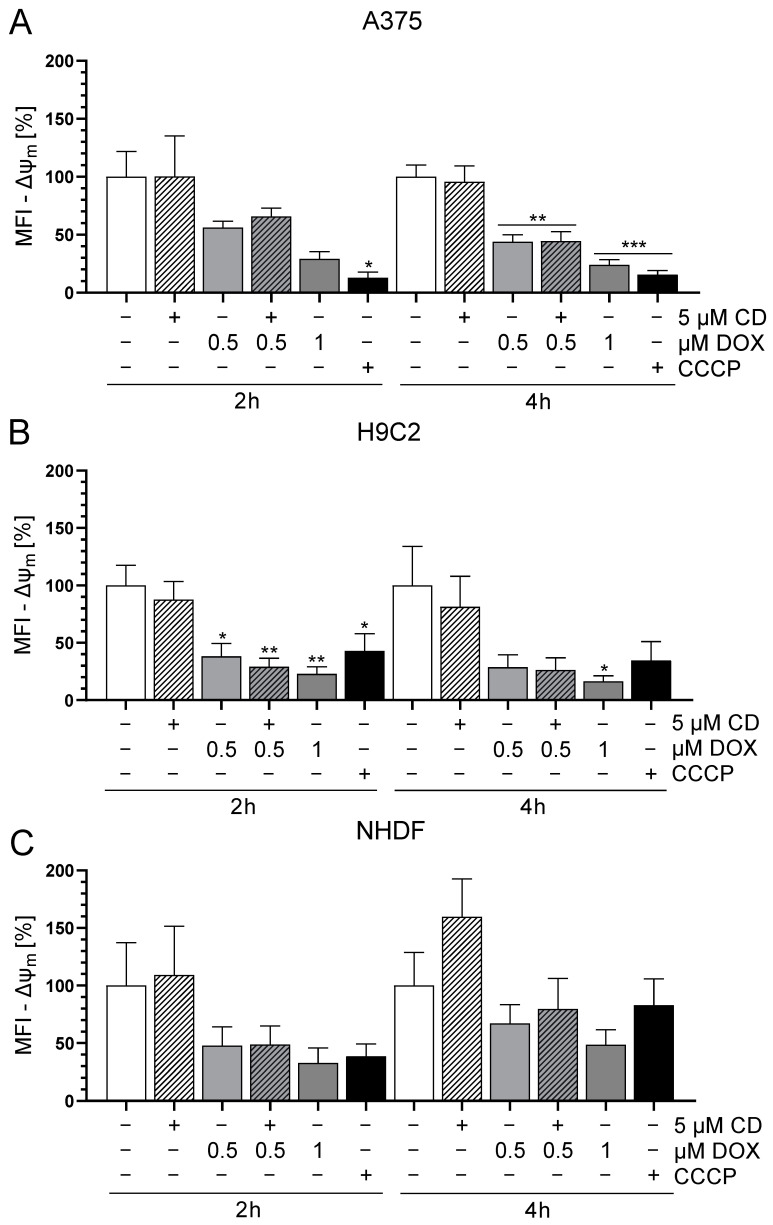
Effect of DOX and CD on the mitochondrial membrane potential (Δψ_m_) in A375, H9C2 and NHDF. (**A**–**C**): A375 (**A**), H9C2 (**B**) and NHDF (**C**) were treated with DOX and CD alone or in combination for 2 or 4 h, followed by tetramethylrhodamine methyl ester (TMRM, 100 nM) staining. CCCP served as positive control. The level of TMRM was determined by flow cytometry using CyFlow Cube 6, and FlowJo software was used to calculate the median fluorescent intensity (MFI). Mock-treated controls were set to 100%. Data represent means ± SEM, *n* ≥ 3. One-way ANOVA was used to determine statistical significance compared to respective control. * *p* ≤ 0.05, ** *p* ≤ 0.01, *** *p* ≤ 0.001.

The treatment with CD had no effect on the Δψ_m_ in any of the cell lines, whereas DOX decreased the Δψ_m_ in all cell lines (Figure 2). In the case of A375 cells, the DOX-induced decrease in Δψ_m_ was time-dependent and was stronger and more significant after 4 h compared to 2 h (Figure 2A). This effect was converse in the normal (healthy) cell line NHDF, where 2 h DOX treatment showed a higher impact compared to 4 h (Figure 2C). As for H9C2 cells, there was a significant drop in Δψ_m_ after all DOX concentrations after 2 h. In case of the 4 h treatment, only 1 µM DOX showed a significant effect but all DOX concentrations seemed to have an impact on the Δψ_m_ (Figure 2B). When comparing all cell types, H9C2 was the most sensitive and resulted in the highest decrease in Δψ_m_ after DOX treatment. Furthermore, there was no difference between the treatment with DOX alone or in combination with CD. Therefore, a change in the Δψ_m_ was most probably not involved in the protective effect of CD seen in normal (healthy) cells, whereas it could have had an impact on the DOX-induced cytotoxicity seen in all cell lines.

### 3.3. CD Treatment Further Increased the High Basal ROS Level in A375 Melanoma Cells

The loss of Δψ_m_ is often associated with the formation of reactive oxygen species (ROS) [45]. Therefore, the basal level of ROS and the impact of CD and DOX on ROS formation was measured using a flow cytometric approach.

**Figure 3:**
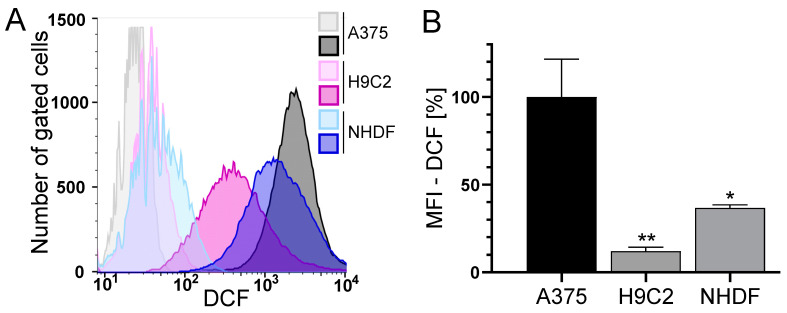
The basal ROS level in A375, H9C2 and NHDF. (**A**,**B**): A375, H9C2 and NHDF were treated with 0.1 µM H_2_DCF-DA and the level of DCF as a measure of intracellular ROS was determined by flow cytometry using a CyFlow Cube 6. FlowJo software was used to create a histogram overlay (**A**) and to calculate the MFI (**B**). (**A**) Representative histograms for treatment with H_2_DCF-DA (dark histograms) were depicted, and unstained cells served as the negative control (light histograms). Fluorescence intensity is plotted against the cell number. (**B**) The MFI of the cells treated with H_2_DCF-DA is depicted. The MFI of unstained cells served as the negative control and the MFI of A375 cells was set to 100%. Data represent means ± SEM, *n* ≥ 3. A one-way ANOVA was used to determine statistical significance compared to the control. * *p* ≤ 0.05, ** *p* ≤ 0.01.

**Figure 4:**
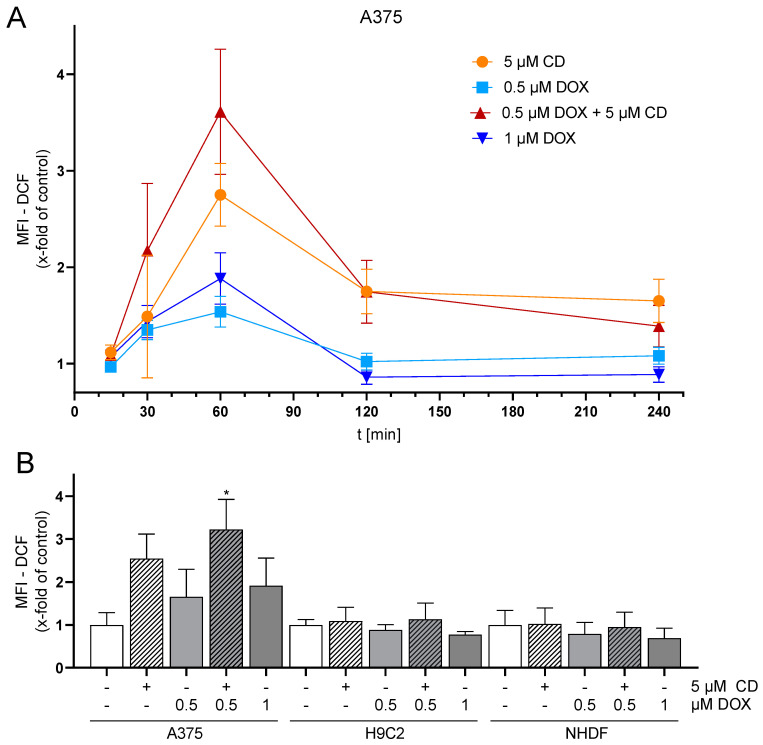
Effect of DOX and CD on ROS production in A375, H9C2 and NHDF. (**A**) A375 cells were preincubated with H_2_DCF-DA (20 µM, 20 min) and treated with DOX alone or in combination with 5 µM CD for 15 min–4 h. (**B**) For comparing A375, H9C2 and NHDF, cells were stained with H_2_DCF-DA (20 µM, 20 min) and treated with DOX alone or in combination with 5 µM CD for 1 h. Level of DCF was determined by flow cytometry using CyFlow Cube 6, and FlowJo software was used to calculate MFI. Mock-treated control was set to 1. Data represent means ± SEM, *n* ≥ 3. One-way ANOVA was used to determine statistical significance compared to respective control. * *p* ≤ 0.05.

The level of basal ROS was significantly higher in the tumor cell line A375 compared to the normal (healthy) H9C2 and NHDF (Figure 3). H9C2 cells only exhibited 10% and NHDF 40% of the ROS compared to A375 cells (Figure 3B). A375 melanoma cells showed a dose- and time-dependent but not significant increase in ROS formation after treatment with DOX. Treatment with CD caused an even higher increase in ROS formation, which was further augmented by the addition of DOX (additive effect, Figure 4). For normal (healthy) cells, a 1 h treatment with CD and DOX was chosen, as a CD- and DOX-induced peak in ROS formation was demonstrated after 1 h treatment in A375 melanoma cells (Figure 4A). No increase in the ROS amount could be detected in the case of normal (healthy) H9C2 and NHDF with the treatment options.

### 3.4. The Combination of Trolox and Catalase or NAC Alone Rescued CD-Induced ROS Formation in Melanoma Cells

**Figure 5:**
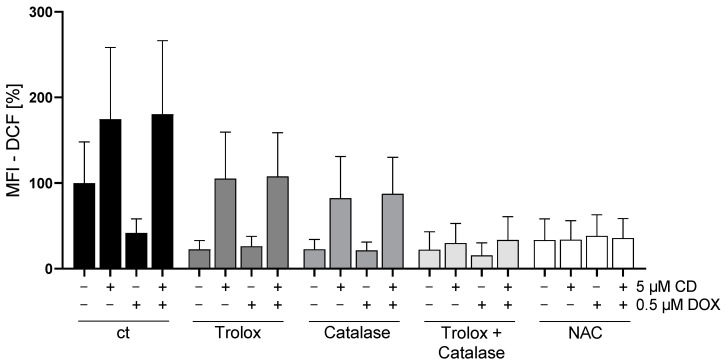
Effect of different antioxidants on DOX- and CD-induced ROS production in A375 cells. A375 cells were stained with H_2_DCF-DA (20 µM, 20 min) and treated with different antioxidants (trolox, catalase or N-acetylcysteine (NAC)) for 4 h, followed by treatment with DOX alone or in combination with 5 µM CD for 1 h. Level of DCF was determined by flow cytometry using CyFlow Cube 6, and FlowJo software was used to calculate MFI. Mock-treated control (cells treated with no drugs and no antioxidant) was set to 100%. Data represent means ± SEM, *n* ≥ 3. Two-way ANOVA was used to determine statistical significance between antioxidant-untreated and -treated conditions.

To validate previous findings (Figure 4), rescue experiments with different antioxidants were performed. As a drug-dependent increase in the ROS level was only measured in A375 tumor cells, only these cells were analyzed (Figure 5).

All antioxidants (trolox, catalase, and the combination or N-acetylcysteine (NAC) alone) showed the tendency to decrease the basal ROS level in A375 cells (ct, -CD, -DOX). When looking at the CD-induced ROS formation, trolox and catalase also slightly decreased the level of ROS, but only the combination of catalase and trolox or NAC alone lowered the ROS amount to the level of the cells treated only with antioxidants (-CD, -DOX).

**Figure 6:**
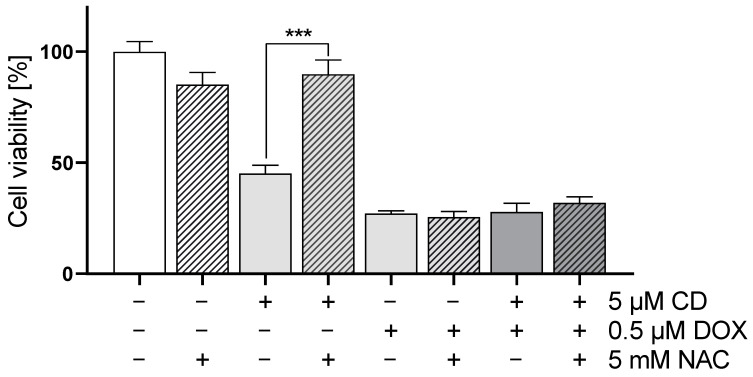
Effect of antioxidant NAC on DOX- and CD-modulated cell viability in A375 cells. A375 cells were treated with 5 mM NAC for 4 h, followed by treatment with DOX alone or in combination with 5 µM CD for 24 h. Cell viability was determined via MTT assay and mock-treated control (ct) was set to 100%. Data represent means ± SEM, *n* = 4. Two-way ANOVA was used to determine statistical significance between NAC-untreated and -treated conditions. *** *p* ≤ 0.001.

In order to evaluate the impact of ROS generation on DOX- and CD-modulated cell viability, rescue experiments were performed (Figure 6). As NAC was able to decrease the CD-induced formation of ROS below the base level (Figure 5; ct, -CD, -DOX), the protective effect of NAC on cell viability in A375 tumor cells was investigated.

The addition of NAC led to a full rescue of the CD-induced cytotoxicity in A375 melanoma cells without affecting the cytotoxicity of DOX alone or the combinational treatment.

### 3.5. CD Is Thiol Reactive and Interacted with NAC via Michael Addition

N-acetylcysteine (NAC), a derivative of the natural amino acid L-cysteine, exerts its antioxidant activity by a direct radical scavenging mechanism or by an indirect mechanism through acting as a cysteine source [46,47]. Cysteine is a component of glutathione, which is an essential intracellular antioxidant. The direct ROS-scavenging effect of NAC is attributed to its thiol group. CD contains an α,β-unsaturated carbonyl group and, thus, is thiol reactive. As NAC showed the most prominent effect in the context of treatment with the antioxidants (Figure 5) and was able to fully rescue CD-induced cytotoxicity (Figure 6), it might be possible that CD directly interacts with NAC. To test this hypothesis, the interaction of CD with NAC was analyzed in a cell-free assay using a UV/Vis spectrophotometer.

**Figure 7:**
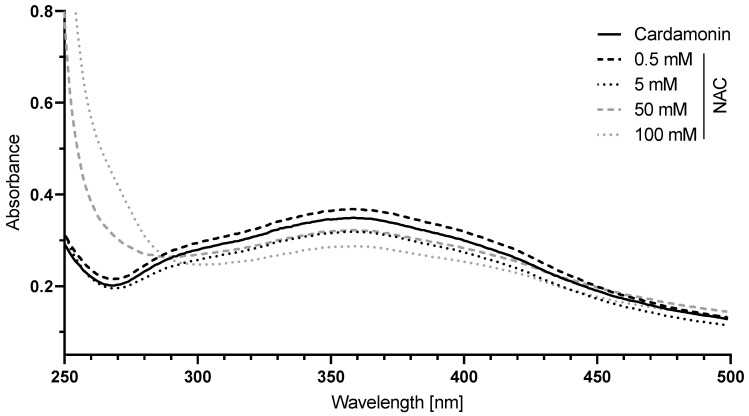
The interaction of N-acetylcysteine (NAC) with CD. A 50 µM CD solution in 0.1 M sodium dihydrogen phosphate buffer was incubated with 0.5 to 100 mM NAC for 10 min and the absorption between 250 and 500 nm was measured. The absorption spectrum of the buffer was used for background correction.

With increasing concentrations of NAC, the absorption spectrum of CD decreased between 290 and 450 nm while increasing before 290 nm (Figure 7), reflecting a decrease in the amount of free CD and an increase in the amount of covalently bound CD to NAC via Michael addition.

To furthermore test the thiol reactivity of CD, the expression of heme oxygenase-1 (HO-1) was tested, which is regulated by the Nrf-Keap1 pathway and used as biochemical marker for thiol-reactive substances as described before [48,49].

**Figure 8:**
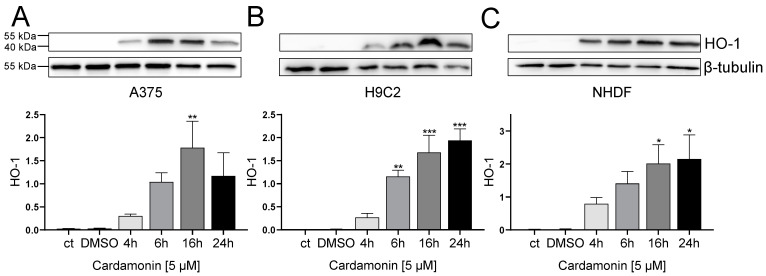
Effect of CD treatment on heme oxygenase 1 (HO-1) abundance in A375, H9C2 and NHDF. (**A**–**C**): Western blotting analysis of HO-1 in A375 (**A**), H9C2 (**B**) and NHDF (**C**) after treatment with 5 µM CD for 4, 6, 16 and 24 h. Densitometric analysis of Western blots was performed using FusionCapt Advance FX 16.09 software. Representative blots and respective protein quantifications in relation to loading control (β-tubulin) are depicted. Medium (ct) and mock-treated control (DMSO) were used as control. Data represent means ± SEM, *n* = 3. One-way ANOVA was used to determine statistical significance compared to control. * *p* ≤ 0.05, ** *p* ≤ 0.01 and *** *p* ≤ 0.001.

CD increased the amount of HO-1 in all cell types in a time-dependent manner. Starting with no HO-1 signal in the medium and solvent control, HO-1 was already upregulated after 4 h, further increasing up to 24 h (Figure 8).

In summary, it could be shown that accessible thiol groups are a target of cardamonin. Moreover, CD caused an upregulation of HO-1 in all cell types (Figure 8) whereas a formation of ROS could only be detected in A375 melanoma cells (Figure 4 and Figure 5). Therefore, the CD-induced upregulation of HO-1 was confirmed to be ROS-independent.

### 3.6. CD Selectively Impaired Mitochondrial Respiration in A375 Tumor Cells

**Figure 9:**
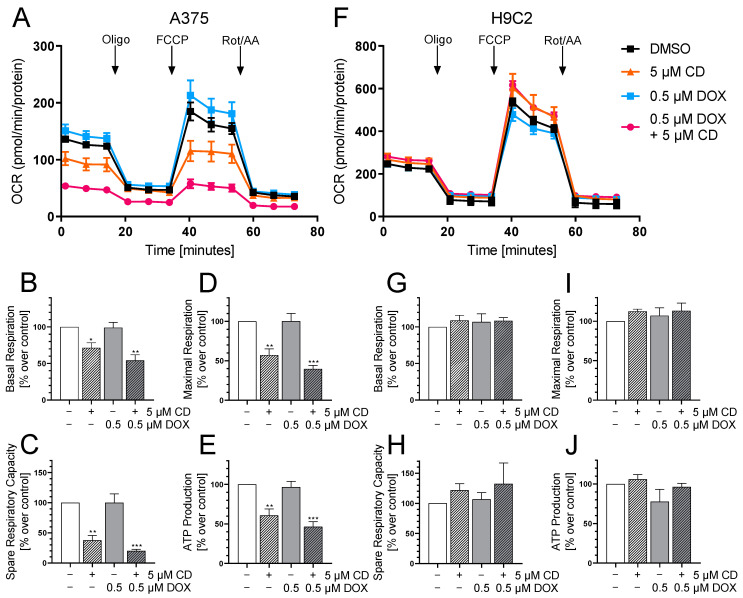
The effect of DOX and CD on mitochondrial respiration in A375 and H9C2 cells. (**A**–**J**): A375 and H9C2 cells were treated with 5 µM CD for 24 h with or without the addition of different concentrations of DOX for the last 4 h. After successive injection of oligomycin (Oligo), FCCP and rotenone/antimycin A (Rot/AA), the oxygen consumption rate (OCR) was measured using a Seahorse XF Analyzer. (**A**,**F**) A representative curve is depicted for A375 (**A**) and H9C2 cells (**F**). (**B**–**E**,**G**–**J**) Based on the OCR in response to the mitochondrial stressors, the parameters “Basal Respiration” (**B**,**G**), “Spare Respiratory Capacity” (**C**,**H**), “Maximal Respiration” (**D**,**I**) and “ATP Production” (**E**,**J**) were calculated for A375 (**B**–**E**) and H9C2 cells (**G**–**J**). The mock-treated control (DMSO) was set to 100%. The data represent means ± SEM, *n* ≥ 3. One-way ANOVA was used to determine statistical significance compared to the respective control. * *p* ≤ 0.05, ** *p* ≤ 0.01, *** *p* ≤ 0.001.

In order to further study the effect of DOX and CD on mitochondria, mitochondrial respiration was measured using a Seahorse XF Analyzer (Figure 9), as energy production by oxidative phosphorylation was described to require an intact Δψ_m_ [50,51].

The Seahorse XF Analyzer measures the oxygen consumption rate (OCR) after the consecutive injection of the mitochondrial stressors oligomycin (Oligo), FCCP and rotenone/antimycin A (Rot/AA). With the help of the measured OCR, different bioenergetic parameters including the basal respiration, maximal respiration, spare respiratory capacity and ATP production can be calculated.

Here, the effect of the drugs alone or in combination on the OCR in A375 tumor cells was compared to H9C2 cardiac myoblasts as the cardiac myoblasts showed a significant DOX-dependent decrease in cell viability at 24 h with the indicated concentration (Figure 9C). A 24 h treatment with DOX and CD already decreased cell viability in A375 melanoma cells drastically, but only fully confluent cells are suitable for measuring the OCR. Therefore, a 24 h CD treatment including a 4 h DOX treatment was chosen.

While DOX showed no impact, CD affected the mitochondrial respiration of A375 tumor cells, significantly decreasing basal respiration, maximal respiration and spare respiratory capacity as well as ATP production (Figure 9A–E). A further drop was induced by the addition of DOX decreasing the basal respiration from 71 (CD alone) to 54% (combination), the maximal respiration from 57 to 40%, the spare respiratory capacity from 38 to 20% and the ATP production from 61 to 47%. DOX seemed to have an additional effect on the CD-impaired respiration while having no effect after 4 h of the single treatment. On the other hand, the respiration of the normal (healthy) H9C2 cells was not affected by CD, DOX or the combinational treatment at the indicated time point (Figure 9F–J).

### 3.7. DOX and CD Selectively Induced Apoptosis in A375 Cells but Not in Normal (Healthy) Cells

Based on the Δψ_m_ data for the tumor cells, the possible involvement of apoptosis was investigated. The cleavage of poly(ADP-ribose) polymerase (PARP) was used as a late apoptotic marker [52,53,54].

**Figure 10:**
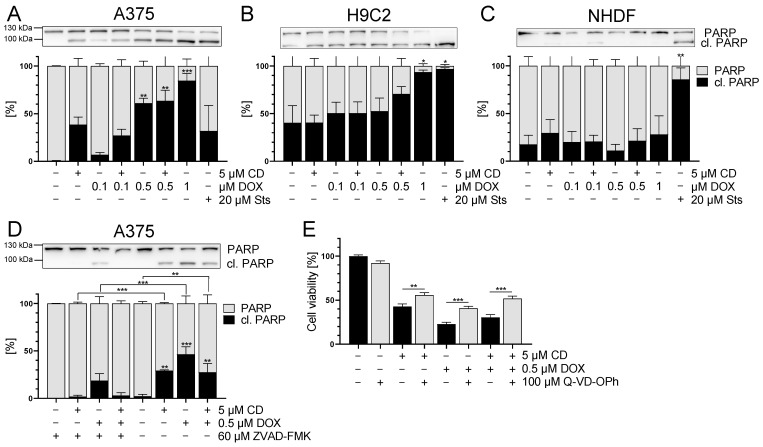
The effect of DOX and CD on apoptosis in A375, H9C2 and NHDF. (**A**–**C**): PARP cleavage (in black) after treatment with DOX alone or in combination with 5 µM CD for 24 h in A375 (**A**), H9C2 (**B**) and NHDF (**C**). Staurosporine (Sts, 20 μM) served as the positive control. (**D**,**E**): A375 cells were treated with the pan caspase inhibitors Z-VAD-FMK (**D**) or Q-VD-OPh (**E**) for 4 h, followed by treatment with DOX and CD alone or in combination for further 24 h. (**A**–**D**) Western blot analysis of PARP cleavage. Densitometric analysis of the Western blots was performed using FusionCapt Advance software and the total amount of PARP and cleaved PARP was set to 100%. Representative blots and the respective quantifications are depicted. (**E**) Cell viability was determined with the MTT assay and the optical density (OD) of the mock-treated control was set to 100%. The data represent means ± SEM, *n* = 3. One-way ANOVA was used to determine statistical significance compared to the control (**A**–**C**). Two-way ANOVA was used to determine the statistical significance between the Z-VAD-FMK- or Q-VD-OPh-untreated and -treated conditions (**D**,**E**). * *p* ≤ 0.05, ** *p* ≤ 0.01, *** *p* ≤ 0.001.

In the A375 cells, PARP cleavage was induced by CD, DOX and the combinational treatment (Figure 10A). The induction of PARP cleavage by DOX treatment was dose-dependent starting at 0.1 µM and significant at concentrations of 0.5 µM DOX. Surprisingly, mock-treated normal (healthy) H9C2 cells already showed a high level of cleaved PARP. A significant increase in PARP cleavage could only be seen after the treatment with 1 µM DOX and in the case of the positive control staurosporine (Sts) (Figure 10B). For normal (healthy) NHDF, no induction of PARP cleavage by CD, DOX or the combinational treatment was observed. Only the positive control Sts resulted in cleaved PARP (Figure 10C).

To confirm that DOX- and CD-initiated apoptosis was responsible for the cytotoxicity in A375 cells, rescue experiments were performed. A375 cells were treated with the pan caspase inhibitor Z-VAD-FMK or Q-VD-OPh, followed by treatment with DOX and CD alone or in combination. The broad-spectrum caspase inhibitor Q-VD-OPh was described as significantly more effective in preventing apoptosis and also being non-toxic, even at higher doses than other inhibitors, especially when testing the cell viability [55]. After 24 h treatment, Western blot analysis of cleaved PARP was performed (Figure 10D), and cell viability was measured via the MTT assay (Figure 10E). Z-VAD-FMK caused a significant decrease of PARP cleavage in all conditions, where cleaved PARP levels were almost at zero, except in the DOX single treatments (Figure 10D). Looking at the cell viability, Q-VD-OPh caused a partial but significant rescue of CD- and DOX-induced single and combinational cytotoxicity (Figure 10E).

These data indicated that apoptosis plays a role in the CD- and DOX-induced cytotoxicity in A375 melanoma cells. In contrast, a combinational treatment of DOX (0.1, 0.5 µM) and CD (5 µM) did not result in an increase of apoptotic cell death. However, there was no full rescue of cell viability with Q-VD-OPh, indicating that other cell death mechanisms might be involved as well.

## 4. Discussion

Treatment options for malignant melanoma still remain unsatisfactory due to the invasive nature and high capability to develop resistance to a variety of different therapies [1,2,11]. The cytotoxic anthracycline doxorubicin (DOX) represents a highly effective chemotherapeutic drug which might be suitable in the treatment of melanomas [7,15,16,56]. Despite its promising anti-tumor activities, DOX therapy exhibits various disadvantages including systemic effects like cardiotoxicity being the most harmful side effect for patients [12,13,14]. Combinational treatments have been studied to either enhance efficacy, promote in situ DOX delivery or to counteract side effects [3,7,8,15,16,19,21].

The use of natural compounds like chalcones represents a promising tool to approach a variety of diseases as they exhibit anti-tumor, anti-oxidative, anti-inflammatory and anti-bacterial activities [25,26,27,28]. For instance, the natural chalcone cardamonin (CD) was demonstrated to protect cells from acute lung injury in sepsis [33] and has a cytotoxic effect on multiple myeloma, colorectal cancer, neuroblastoma and melanoma cells [32,34,35,36]. In this in vitro study, we analyzed the efficacy of the combinational treatment of the classical chemotherapeutic DOX with the natural compound CD. As a representative tumor cell line, A375 human skin melanoma cells were used. As a model for healthy cells, normal dermal fibroblasts (NHDF) were studied, and healthy rat cardiac myoblasts (H9C2 cells) were used to demonstrate DOX cardiotoxicity.

Several studies revealed the unselective cytotoxicity of DOX treatment [12,19,57]. DOX significantly decreased cell viability in melanoma as well as in normal (healthy) cells in a dose- and time-dependent manner. The lowest effect was seen in fibroblasts with an IC_50_ value of 0.86 μM and the highest in A375 cells with an IC_50_ value of 0.08 μM and in H9C2 cells with 0.12 μM. These results validated the unselective effect of DOX in cardiac myoblasts and reflected the vulnerability of cardiac cells to DOX treatment and thus cardiotoxicity [10,12,13,14]. On the other hand, CD was very effective in decreasing the cell viability of melanoma cells with an IC_50_ value of 2.76 μM, whereas normal (healthy) cells were not affected. The selective mechanism of action of CD was already demonstrated by Berning et al. in A375 melanoma cells [36] and by Wenzel et al. in neuroblastoma cells [35].

In the combinational treatment, CD protected normal cardiac myoblasts and dermal fibroblasts from DOX-induced toxicity. In the case of 0.1 μM DOX, the addition of CD caused a significant increase in cell viability from 42 to 96% in H9C2 cardiac myoblasts and from 85 to 129% in NHDF. The protective qualities of CD and other natural chalcones were demonstrated before. For example, CD exhibited a protective effect from acute lung injury [33] and from DOX-initiated cardiotoxicity in vitro and in vivo [29]. Qi et al. found that cardamonin might be a potential activator of the transcription factor Nrf2, preventing its degradation with subsequent upregulation of heme oxygenase 1 (HO-1), glutathione (GSH), superoxide dismutase (SOD) and catalase. The antioxidant response mitigated oxidative stress and inhibited apoptotic processes in vitro and in vivo [29]. However, tumor cells were not taken into consideration in their interesting study. The natural chalcone pulichalconoid B attenuated the accumulation of ROS and protected astrocytes from ROS-induced apoptosis [58]. The chalcone derivative linearthin protected skin cells from ROS generated by UVB exposure [59], and the chalcone butein prevented glutamate-induced oxidative damage in neuronal cells [60]. Moreover, the combination of DOX and the natural chalcone naringenin reversed the DOX-induced downregulation of antioxidant enzymes and upregulation of inflammatory molecules in vivo [21]. The exposure of A375 melanoma cells to the combinational treatment significantly decreased cell viability compared to the mock-treated control.

Consequently, the unselective cytotoxicity of DOX as well as the selective cytotoxicity of CD on melanoma cells, fibroblasts and cardiac myoblasts was demonstrated. On top of that, a protective effect of CD on DOX-induced toxicity in normal (healthy) cells was observed, whereas the combinational treatment still exhibited cytotoxic effects in melanoma cells. To further characterize the combination of DOX and CD, a possible underlying mechanism of cell death was investigated.

The induction of apoptosis by DOX and CD single treatment was demonstrated in several studies and various cells lines [32,34,35,36,57,61]. In fact, CD as single drug was already shown to be cytotoxic and to induce apoptosis in A375 melanoma cells [36]. However, the involvement of apoptosis or other cell death mechanisms in the combinational treatment, especially with respect to the protective effect of CD on DOX-induced toxicity in normal (healthy) cells, has yet to be determined. Hence, we studied the single and combinational treatment of DOX and CD in melanoma cells, dermal fibroblasts, and cardiac myoblasts in order to determine the mechanism of cell death.

The activation of cell death mechanisms often correlates with changes in the mitochondrial membrane potential (Δψ_m_). A decrease in Δψ_m_ has been described to be associated with early targets of apoptosis and to be an early marker of apoptosis [43,44]. DOX treatment resulted in a dose-dependent decrease in Δψ_m_ in all cell lines. The effect of DOX on the Δψ_m_ was the highest on the rat cardiac myoblast with a similar effect on the A375 melanoma cells, but a lower effect on NHDF. In contrast, CD did not modify the Δψ_m_ in any of the cells. There was neither an effect of the single treatment nor an additional or protective effect in the combinational treatment. Wenzel et al. described a selective decrease of the Δψ_m_ after CD in SH-SY5Y human neuroblastomas starting at 6 h [35]. Other groups have shown a decrease by CD after 9 h [62] and 48 h [63]. A possible effect of CD on the Δψ_m_ after extended time points cannot be excluded, but a protective effect of CD on the DOX-induced decrease in Δψ_m_ in the normal (healthy) cells could be excluded. Therefore, changes in the Δψ_m_ have not been involved in the protective effect of CD on DOX-induced cytotoxicity in normal (healthy) cells. However, the decrease of Δψ_m_ after DOX treatment might impact the DOX-induced cytotoxicity seen in all cell lines.

Cancer cells usually exhibit higher levels of basal reactive oxygen species (ROS) due to changes in their metabolism [64]. In this study, melanoma cells displayed the highest basal ROS level, compared to normal (healthy) cardiac myoblasts and fibroblasts. As ROS are often associated with a loss in Δψ_m_ and might cause apoptosis [45], the effect of DOX, CD and the combinational treatment on ROS formation was tested. While DOX caused a slight but dose-dependent increase in ROS in melanoma cells, CD-induced ROS levels were even higher. A significant and additive increase was achieved with the combinational treatment. While DOX-induced ROS formation was demonstrated in several studies [12,21,61,65], a significant increase in melanoma cells was not detected, at least with the indicated concentrations and 1 h treatment. DOX has been shown to generate superoxide by redox cycling at its (semi)quinone structure. This superoxide can be converted to hydrogen peroxide by the superoxide dismutase (SOD) within the cell, which in turn can be catalyzed to the hydroxyl radical by free iron via the Fenton reaction [66]. However, the used DCF assay only measures hydrogen peroxide, hydroxyl radicals, hydroperoxides and peroxynitrite [40,67]. Superoxide generated by DOX might not have been detected by the DCF assay. Nevertheless, N-acetylcysteine (NAC), a potent antioxidant, was not able to rescue the DOX-induced cytotoxicity, demonstrating that the DOX toxicity in A375 melanoma cells is expected to be ROS-independent. Even though a ROS-independent apoptosis in A375 melanoma cells was shown earlier [36], the ROS-dependent cytotoxicity of CD treatment was demonstrated in other studies [34,62]. Although DOX-induced ROS formation was described in H9C2 cardiac myoblasts [61], normal (healthy) cells were not affected by DOX in this study. To further analyze ROS formation in melanoma cells, rescue experiments with different antioxidants were performed. Trolox, a vitamin E derivative which prevents oxidative stress caused by peroxynitrite [68], decreased basal ROS levels but did not protect melanoma cells from CD-induced ROS. Similar results were obtained by the use of catalase, an enzyme protecting cells from oxidative damage by converting hydrogen peroxide to water and oxygen [69]. While single pretreatment showed no effect, the combinational treatment of trolox and catalase diminished the CD-induced ROS formation. Furthermore, the most commonly used antioxidant N-acetylcysteine (NAC) was able to reduce basal ROS levels as well as CD-induced ROS formation. As NAC was shown to exert its antioxidant activity by different mechanisms [46,70], precise species of ROS cannot be identified just by the ROS-scavenging effect of NAC itself. However, the scavenging properties of trolox and catalase demonstrated that a basal as well as a CD-induced level of hydrogen peroxide and/or peroxynitrite could be involved. Nevertheless, other species of ROS might also be involved.

Cardamonin contains an α,β-unsaturated carbonyl group and its biological activity is associated with its thiol reactivity [48]. Indeed, CD was able to directly interact with NAC via Michael addition in a cell-free assay, thus proving that accessible thiol groups are targets of CD. Furthermore, NAC was able to fully rescue CD-induced ROS formation as well as its cytotoxicity. Therefore, the cytotoxic effect of CD on melanoma cells was associated with CD-induced ROS formation and/or its thiol reactivity.

Moreover, as CD was demonstrated to interact with NAC, a derivative of the natural amino acid L-cysteine, it might have an impact on the level of glutathione (GSH). GSH is an essential intracellular antioxidant and is often overexpressed in cancer cells [71]. Its synthesis consists of two reactions: (1) the synthesis of L-glutamate and L-cysteine to form γ-glutamylcysteine and (2) the addition of glycine. As the first reaction is the rate-limiting step of the GSH synthesis, a depletion of L-cysteine might affect the overall GSH levels [72]. The α,β-unsaturated carbonyl group of cardamonin was shown to interact with the thiol group of NAC in a cell-free assay and might be able to interact with L-cysteine within a cell as well. Thus, CD might interact with glutathione itself or with L-cysteine preventing the formation of additional GSH. As cancer cells usually exhibit higher levels of basal ROS [64] as also demonstrated with the melanoma cells used in this study, an elevated GSH level might protect them from oxidative damage. If CD is able to deplete GSH levels, the GSH is no longer able to protect tumor cells from excessive amounts of ROS produced, leading to oxidative damage and ultimately cell death. Thus, the CD-mediated increase in the ROS amount in melanoma cells might also be an indirect effect. On the other hand, normal (healthy) cells might not be affected by a depletion of GSH as they exhibit lower basal ROS levels and have a higher tolerance towards ROS.

A biochemical parameter being upregulated by ROS is HO-1 [49,73], being a prominent marker for oxidative stress. However, HO-1 can also be ROS-independently expressed, if thiol reactive compounds such as CD are available [27,49]. In this study, a CD-initiated upregulation of HO-1 was shown for all cell types studied, even though an upregulation of ROS was interestingly only seen in A375 cells. A ROS-independent HO-1 induction by CD was shown in A375 melanoma cells and NHDF before [36]. Furthermore, CD was demonstrated to directly interact with Kelch-like ECH-associated protein 1 (Keap1) which leads to the nuclear translocation of Nuclear Factor Erythroid 2-related Factor 2 (Nrf2) and subsequent upregulation of HO-1 [74]. An upregulation of HO-1 is often accompanied by the upregulation of, for example, antioxidant enzymes and GSH synthesis in parallel as the common Nrf2/ARE signaling axis will be used. As an example, in addition to the upregulation of HO-1 also SOD, GPx1, and catalase were expressed after the treatment of HL-1 cardiomyocytes with cardamonin [29]. Therefore, we may speculate that it could also work in our study, but indeed we did not check it herein. Several studies described the cytoprotective effects of HO-1 in various diseases which were associated with the reduction of oxidative stress, inflammation and maintaining mitochondrial integrity [37,74,75,76]. Interestingly, the curcuminoid curcumin protected H9C2 cardiac myoblasts from hydrogen-peroxide-induced apoptosis via the upregulation of HO-1, proving the protective capabilities of HO-1 against oxidative stress [75]. Additionally, HO-1 might exhibit anti-tumor activities as its overexpression reduced tumor size and resulted in a longer overall survival rate of breast cancer patients [37]. Even though overexpression of HO-1 in melanoma cells resulted in higher proliferation, angiogenic potential, metastasis and a decreased survival of mice [77], a systemic HO-1 overexpression caused reduced growth and metastasis of melanoma in mice, triggered by the HO-1 overexpression in tumor-infiltrating immune cells [78].

A physiological Δψ_m_ is essential for the generation and storage of energy, and a decrease might lead to dysfunctional mitochondria and energy depletion [50]. A DOX-induced decrease in Δψ_m_ could result in decreased respiration and ATP production. Even though CD had no influence on the Δψ_m_ at least after 4 h, it significantly decreased the basal respiration, maximal respiration, spare respiratory capacity and ATP production in melanoma cells. In contrast, in triple negative breast cancer cells, CD significantly increased the basal respiration, maximal respiration and spare respiratory capacity after 6 h [62]. CD-impaired respiration was further lowered by the addition of DOX. Normal rat cardiac myoblasts were neither affected by CD or DOX single treatment nor by the combinational treatment at the studied time points. As opposed to this, other groups have shown a DOX-induced decrease in mitochondrial respiration in H9C2 cardiac myoblasts after treatment with higher concentrations and a longer period of time [65]. Taken together, DOX did not affect the respiration of melanoma cells or rat cardiac myoblasts, at least after a short treatment of 4 h. Interestingly, the CD-impaired respiration in melanoma cells was further decreased by the addition of DOX.

To confirm the Δψ_m_ data and to further analyze the involvement of apoptosis in the unselective DOX cytotoxicity and selective toxicity of CD single and combinational treatment, a late apoptotic marker, namely PARP, was studied. After the initiation of apoptosis, a variety of caspases are activated, which subsequently cleave a series of different substrates. For instance, activated caspase-3 and -7 cleave poly(ADP-ribose) polymerase (PARP), resulting in cleaved PARP, which is a hallmark for apoptosis [79]. CD increased PARP cleavage in A375 melanoma cells while having no effect on normal (healthy) cells. DOX treatment increased the cleavage of PARP in a dose-dependent manner. As there was no synergistic or additive effect caused by the addition of CD, a similar effect of CD and DOX on apoptosis could be assumed. However, the pan caspase inhibitor Z-VAD-FMK did not fully rescue PARP cleavage in DOX-treated A375 cells. Even though the broad-spectrum caspase inhibitor Q-VD-OPh significantly increased cell viability, it did not fully rescue the cytotoxic effects of DOX or CD single treatment nor the combinational treatment. Therefore, other cell death mechanisms might be involved. On the other hand, cardiac myoblasts already displayed high levels of cleaved PARP in the mock-treated control, and only exhibited significant PARP cleavage after 1 µM DOX. High basal levels of cleaved PARP in H9C2 cells were observed by other groups as well [57,80] and might cause insensitivity of the assay. Interestingly, fibroblasts were not affected by any of the treatment conditions, except by the positive control staurosporine (Sts). Thus, CD did not protect normal (healthy) cells from DOX-induced apoptosis and did not further increase apoptosis in melanoma cells. However, as a result of the low DOX concentration in the combinational treatment, less apoptosis was induced in normal (healthy) cells compared to higher concentrations of DOX.

## 5. Conclusions

In this in vitro study, we demonstrated the beneficial aspect of combinational treatment with the standard chemotherapeutic agent doxorubicin (DOX) and the natural chalcone cardamonin (CD). DOX decreased cell viability in all cell types. Rat cardiac myoblasts were particularly affected, reflecting DOX-induced cardiotoxicity. In contrast, selective CD-induced cytotoxicity was only seen in A375 melanoma cells, not in normal (healthy) cells. The addition of CD to DOX treatment protected normal (healthy) fibroblasts (NHDF) and cardiac myoblasts from DOX-induced cytotoxicity while still exhibiting the anti-tumor effect in melanoma cells. Moreover, CD was able to directly interact with N-acetylcysteine (NAC) in a cell-free assay and to induce heme oxygenase-1 (HO-1) in a reactive oxygen species (ROS)-independent manner. Furthermore, DOX decreased the mitochondrial membrane potential (Δψ_m_) in all cell types, while CD selectively decreased mitochondrial respiration in A375 melanoma cells, but not in healthy cardiac myoblasts. The DOX-induced cytotoxicity seen in melanoma cells was ROS-independent, whereas the cytotoxic effect of CD was associated with CD-induced ROS-formation and/or its thiol reactivity. All treatments (DOX, CD and the combination) induced apoptosis in A375 melanoma cells. However, the combinational treatment did not induce apoptosis in the normal, not transformed cell types NHDF and H9C2. In conclusion, the combination of two substances with different mechanisms of action, attacking tumor cells from different angles, is possibly a good alternative leading to less resistance. Future experiments with other tumor cell lines dealing with DOX/CD treatment and, in addition, the use of a mouse model should substantiate and consolidate the hypothesis which has been formulated.

## Data Availability

Data are available in a publicly accessible repository.
